# Master regulator NtrC controls the utilization of alternative nitrogen sources in *Pseudomonas stutzeri* A1501

**DOI:** 10.1007/s11274-021-03144-w

**Published:** 2021-09-15

**Authors:** Zhimin Yang, Qin Li, Yongliang Yan, Xiubin Ke, Yueyue Han, Shaoyu Wu, Fanyang Lv, Yahui Shao, Shanshan Jiang, Min Lin, Yunhua Zhang, Yuhua Zhan

**Affiliations:** 1 Biotechnology Research Institute, Chinese Academy of Agricultural Sciences, Beijing, China; 2 School of Resources and Environment, Anhui Agricultural University, Hefei, China

**Keywords:** *Pseudomonas stutzeri*, NtrC, Nitrogen metabolism, RNA-seq

## Abstract

*Pseudomonas stutzeri* A1501 is a model strain used to study associative nitrogen fixation, and it possesses the nitrogen regulatory NtrC protein in the core genome. Nitrogen sources represent one of the important factors affecting the efficiency of biological nitrogen fixation in the natural environment. However, the regulation of NtrC during nitrogen metabolism in *P. stutzeri* A1501 has not been clarified. In this work, a phenotypic analysis of the *ntrC* mutant characterized the roles of NtrC in nitrogen metabolism and the oxidative stress response of *P. stutzeri* A1501. To systematically identify NtrC-controlled gene expression, RNA-seq was performed to further analyse the gene expression differences between the wild-type strain and the ∆*ntrC* mutant under nitrogen fixation conditions. A total of 1431 genes were found to be significantly altered by *ntrC* deletion, among which 147 associative genes had NtrC-binding sites, and the pathways for nitrogen fixation regulation, nitrogenous compound acquisition and catabolism and nitrate assimilation were discussed. Furthermore, the oxidative stress-related gene (*katB*), which was upregulated by *ntrC* deletion, was suggested to be a potential target gene of NtrC, thus highlighting the importance of NtrC in nitrogenase protection against oxygen damage. Based on these findings, we propose that NtrC is a high-ranking element in the regulatory network of *P. stutzeri* A1501 that controls a variety of nitrogen metabolic and oxidative stress responsive traits required for adaptation to complex rhizosphere environments.

**Supplementary Information:**

The online version contains supplementary material available at 10.1007/s11274-021-03144-w.

## Introduction

Nitrogen is one of the most important limiting elements in the environment. Bacteria have evolved many systems to adjust their cell metabolic systems according to the environmental nitrogen supply (Shimizu 2016). NtrC, a regulator protein of nitrogen metabolism, is ubiquitous in bacteria and represents the global regulator of gene expression in response to nitrogen limitation (Jiang and Ninfa 1999, 2009; Schumacher et al. 2013). In bacteria, NtrC and NtrB constitute a two-component regulatory system of nitrogen metabolism, which is mediated by protein phosphorylation signal conduction (Arcondéguy et al. 2001; Ninfa and Jiang 2005). Once phosphorylated, NtrC binds DNA at specific promoters and activates the transcription of target genes (Weiss et al. 1992; Chen and Reitzer 1995).

Homologues of *ntrC* genes have been found in many nitrogen-fixing bacteria, but the role of NtrC in nitrogen fixation is not essential. NtrC activates the transcription of *nifLA* in *Klebsiella pneumoniae* (Merrick 1983; Minchin et al. 1989); however, in *Azotobacter vinelandii*, *Bradyrhizobium japonicum*, and *Azospirillum brasilense*, NtrC is not involved in the expression of *nif* gene expression but plays a role in other aspects of nitrogen assimilation, such as nitrate utilization and glutamine synthase (GS) activity (Toukdarian and Kennedy 1986; Martin et al. 1988; Liang et al. 1993). In addition, the nitrogen assimilation control gene *nac* of *K. pneumoniae* is also regulated by NtrC (Collins et al. 1993). In *Rhodobacter capsulatus*, NtrC is necessary for urea utilization (Masepohl et al. 2001). The NtrC protein was also found to regulate the biosynthesis of alginate, lipase, and biofilms (Leech et al. 2008; Krzeslak et al. 2008; Kim et al. 2009; Cheng et al. 2018). In both *Pseudomonas aeruginosa* and *Pseudomonas fluorescens*, NtrBC and CbrAB form a network to control the C/N balance (Li and Lu 2007; Zhang and Rainey 2008; Sánchez et al. 2017).

*Pseudomonas stutzeri* A1501was isolated from the rice rhizosphere in China and is a model strain for studying associative nitrogen fixation (Desnoues et al. 2003; Rediers et al. 2003; He et al. 2008). This strain has specific nitrogen metabolic properties, including nitrogen fixation under microaerophilic conditions, denitrification under anaerobic conditions and nitrification under aerobic conditions (Lalucat et al. 2006). The regulation of nitrogen fixation (*nif* genes) depends on the general nitrogen regulatory system in the core genome (NtrBC and related genes) and the *nif*-specific regulatory system (NifLA) acquired by horizontal transfer in *P. stutzeri* A1501 (Yan et al. 2008). In addition, because the associated nitrogen-fixing bacteria and root system could not form root nodules and other special tissue structures, *nif* gene expression is also greatly affected by environmental factors. It is conceivable that the protein NtrC may participate in the expression of the regulator NifA in *P. stutzeri* (Xie et al. 2006). However, the role and mechanism of NtrC in the nitrogen metabolism of *P. stutzeri* A1501 have not been clarified clearly. In this study, we provided evidence that NtrC is required for the regulation of nitrogen metabolism and environmental adaption of *P. stutzeri* A1501, including nitrogen compound utilization, nitrate assimilation, denitrification and nitrogen fixation, as well as the oxidative stress response. Furthermore, we utilized RNA-seq to compare the global expression profile of the *ntrC* deletion mutant to the wild type under nitrogen fixation conditions and identified some potential targets regulated by NtrC, such as genes involved in nitrogen fixation, assimilatory nitrate reduction, urea metabolism, electron transport and ammonium transport, demonstrating that NtrC is a global regulator controlling the nitrogen availability of *P. stutzeri* A1501 under nitrogen fixation conditions. Given the global regulatory role of NtrC in nitrogen-fixing bacteria, A1501 NtrC will be a promising element for studying the synthetic biology of nitrogen fixation systems and enhancing the nitrogen-fixing efficiency of root-associated diazotrophs.

## Materials and methods

### Bacterial strains and culture conditions

The bacterial strains and plasmids used in this study are listed in Table 1. Bacteria were cultivated at 30 °C (*Pseudomonas stutzeri*) or 37 °C (*Escherichia coli*) in Luria-Bertani (LB) or mineral lactate medium (medium K). The mineral lactate medium (g L^−1^) contained KH_2_PO_4_ (0.4), K_2_HPO_4_ (0.1), NaCl (0.1), MgSO_4_·7H_2_O (0.2), MnSO_4_·H_2_O (0.01), Fe_2_(SO_4_)_3_·H_2_O (0.01), Na_2_MoO_4_·H_2_O (0.01), C_3_H_5_NaO_3_ (6 mL), and (NH_4_)_2_SO_4_ (0.4). When required, nitrogen sources were supplemented at the following final concentrations: 10 mmol L^−1^ (NH_4_)_2_SO_4_, 10 mmol L^−1^ serine, 10 mmol L^−1^ urea or 10 mmol l^−1^ KNO_3_. Cultivation of the strain under anaerobic conditions was carried out using LB medium supplemented with 10 mM KNO_3_ as a terminal electron acceptor of denitrification. The cultivation vessel, which was 60 mL in volume and contained 20 mL of medium, was sealed with butyl rubber, and the remaining oxygen was removed by flushing the tubes with argon for 10 min. The cultivation vessels were inoculated in triplicate with equal amounts of A1501/A1511 cells and shaken at 200 rpm at 30 °C. At regular time intervals, the OD_600_ of the 200 µL samples was analysed. Antibiotics were used at the following concentrations: 100 µg mL^−1^ ampicillin (Amp); 50 µg mL^−1^ kanamycin (km); 10 µg mL^−1^ tetracycline (Tc); and 40 µg mL^−1^ chloramphenicol (Cm).

**Table.1:** Strains and plasmids used in this study

Strain/plasmid	Relevant characteristics	Source
*P. stutzeri* strains		
A1501	Wild-type, Culture Collection: CGMCC 0351	Lab collection
A1511	*ntrC* deletion mutant, Cm^r^	This study
A1512	A1511 containing pLA*ntrC*, Tc^r^ and Cm^r^	This study
A1513	A1511 containing pLA*cbrB*, Tc^r^ and Cm^r^	This study
*E. coli strains*		
JM109	Competent cell for cloning	Takara
Plasmids		
pLAFR3	Mobilizable vector, Tc^r^	(Staskawicz et al. 1987)
pKatCAT5	Source of the chloramphenicol resistance cassette, Cm^r^	Lab collection
pLA*ntrC*	pLAFR3 derivative carrying the A1501 WT *ntrC* gene under the control of its endogenous promoter, Tc^r^	This study
pLA*cbrB*	pLAFR3 derivative carrying the A1501 WT *cbrB* gene under the control of its endogenous promoter, Tc^r^	This study
pK18*mobSacB*	Allelic exchange vector, Km^r^	(Schäfer et al. 1994)
pk18/del*ntrC*	pK18*mobsacB* derivative carrying a *Bam* HI/*Hin* dIII fragment for homologous recombination, Cm^r^, Km^r^	This study
pRK2013	Helper plasmid for conjugation into *P. stutzeri* A1501, Km^r^	(Figurski and Helinski 1979)
pMD18-T	2.96 kb cloning vector, Amp^r^	Takara

### **Construction of the*ntrC*deletion mutant and complementation plasmids**

For *ntrC* gene replacement, a *sacB*-based strategy was employed (Schäfer et al. 1994). To construct the *ntrC*-null mutant (A1511), amplification of a 772 bp DNA fragment located upstream of *ntrC* was performed using the primer set upF/upR, and amplification of an 806 bp DNA fragment located downstream of *ntrC* was performed using the set downF/downR (Table S1). Restriction enzyme sites (*Bam* HI and *Hin* dIII) incorporated into the oligonucleotide primers to facilitate vector construction are underlined in the oligonucleotide sequences shown in Table S1. An 882 bp DNA fragment containing the Cm resistance cassette was amplified from the plasmid pKatCAT5 by PCR using the primers CmF and CmR. The three amplicons were fused into a 2.46 kb fragment, in which the Cm gene was located between the other two amplicons by overlap extension PCR according to the PCR-based fusion strategy (Shevchuk et al. 2004). The fusion PCR product was then cloned into the multiple cloning sites of the pMD18-T vector (TaKaRa, Japan). The resulting plasmid DNA was double digested with *Bam* HI/*Hin* dIII and then cloned into the *Bam* HI/*Hin* dIII sites of pK18*mobsacB* (Schäfer et al. 1994). The resulting plasmid, pK18/del*ntrC*, was mobilized from *E. coli* into *P. stutzeri* A1501 by conjugation using pRK2013 (Figurski and Helinski 1979) as the helper plasmid. After mating, the cells were spread on LB plates containing 50 µg/mL Km and 40 µg/mL Cm to screen for clones in which pK18/del*ntrC* had integrated into the A1501 genome via a single recombination event. Another recombination event was then induced to replace *ntrC* with *cat* and to remove the Km^r^ and *sacB* genes from the genome. A colony of a single recombinant was then grown in nonselective LB medium at 30 °C. Cultures were diluted and spread onto LB agar supplemented with 10% (wt/vol) sucrose and 40 µg/mL Cm. The *ntrC* mutant strain was selected for the kanamycin-sensitive and *sacB*-negative colonies. Correct recombination was checked using the primers testF and testR, followed by nucleotide sequencing of the amplicon obtained. The resulting *ntrC* deletion mutant, A1511, was used for further study.

DNA fragments containing WT genes for *ntrC* or *cbrB* with their promoter and terminator regions were amplified by PCR to construct complementation plasmids. Two complementation DNA fragments containing *ntrC* or *cbrB* were doubly digested with *Hin* dIII/*Bam* HI and then ligated into the broad host plasmid pLAFR3 (Staskawicz et al. 1987). The resulting two complemented plasmids, pLA*ntrC* and pLA*cbrB*, were used for further studies.

### RNA isolation for qRT–PCR

Total RNA was isolated from bacteria cultured under the described conditions using the SV total RNA isolation system (Promega, Madison, WI) according to the manufacturer’s instructions. Total RNA was quantified using microspectrophotometry (NanoDrop Technologies, Inc.). RNA integrity was measured using an Agilent 2100 Bioanalyser (Agilent Technologies, Inc.). RNA samples with RNA integrity numbers (RINs) above 7.0 and threshold cycle (CT) values above 32 were used for qRT–PCR.

### Quantitative real-time PCR

The expression levels of selected genes were determined by qRT–PCR with Power SYBR green PCR master mix using an ABI Prism 7500 sequence detection system (Applied Biosystems, USA) according to the manufacturer’s instructions. Primers were designed based on sequences of selected genes, which were imported into OligoPerfect (Invitrogen, USA), a primer design software program designed to generate primer pairs suitable for real-time PCR. Primers used for qRT–PCR are listed in Table S1. All qRT–PCRs were performed in triplicate using a 25-ml mixture containing cDNA (5 ml of a one-fifth dilution), 1× brilliant SYBR green quantitative PCR master mixture (Stratagene, USA), and approximately 5 pmol of each primer. Amplification and detection of specific products were performed using the following procedure: 95 °C for 10 min, followed by 40 cycles of 95 °C for 30 s, 55 °C for 1 min, and 72 °C for 30 s and then a dissociation curve analysis. The 16 S rRNA gene was used as the endogenous reference control, and relative gene expression was determined using the 2^−^^ΔΔCT^ relative quantification method. To obtain a standard curve for real-time PCR (RT–PCR), PCR was performed with each primer set by using calibrated amounts of chromosomal DNA, and these assays were performed at the same time as qRT–PCR.

### Nitrogenase activity assays

Nitrogenase activity was determined according to the previously described derepression protocol (Desnoues et al. 2003). Bacterial suspensions were incubated at an OD6_00_ of 0.1 in N-free minimal lactate medium (0.5% oxygen and 10% acetylene) at 30 °C. Protein concentrations were determined using a standard protein assay (Bio–Rad, Hercules, CA) with bovine serum albumin as a standard. The specific activity of nitrogenase was expressed as nmol ethylene per hour per milligram of protein. Each experiment was repeated at least three times.

## RNA-seq

Strains A1501 and A1511 were cultured for 5 h under nitrogen fixation conditions. RNA was extracted using TRIzol LS reagent (Invitrogen, USA) following the manufacturer’s instructions. Host-cell RNA was depleted using a MICROBEnrich kit (Ambion, USA), and bacterial 23 and 16 S rRNAs were subsequently depleted with a MICROBExpress bacterial mRNA enrichment kit (Ambion, USA). Total RNA-seq libraries were then constructed and sequenced using an Illumina HiSeq 2500 instrument and the paired-end method by Tianjin Biochip Corporation (Tianjin, China). The raw tag sequence data were analysed for gene annotation, genome annotation, and functional annotation. The quality of all steps was controlled in accordance with the recommendations of Illumina.

## Transcriptome data analysis

To analyse the gene expression variation of different samples, the fragments per kb of CDS per million mapped reads (FPKM) value was used to normalize the data and represent the overall gene expression. The differentially expressed genes between the two samples were selected according to their significance based on chi-square tests (P < 0.05, with Bonferroni correction) and at least 2-fold differences. Each transcriptome experiment was repeated independently three times (biological replicates).

## Bioinformatics analysis

For the phylogenetic analysis, the amino acid sequences of NtrC proteins from different organisms were obtained from the NCBI. Multiple sequence alignments of full-length proteins were performed using Clustal X (Crooks et al. 2004). The pairwise deletion option was used to circumvent the gaps and missing data. We used the neighbour-joining tree generated by the MEGA (Molecular Evolutionary Genetics Analysis) program with 1000 replicates of bootstrap analysis (Datsenko and Wanner 2000).

For the WebLogo analysis, MEME (http://meme.sdsc.edu/) (Bailey and Elkan 1994) and BioProspector (http://robotics.stanford.edu/~xsliu/BioProspector/) (Liu et al. 2001) were used to perform a sequence analysis of the upstream regions of significantly changed genes. The sequence logo was created with WebLogo (http://weblogo.berkeley.edu/) (Crooks et al. 2004).

### Phenotype microarray (PM) analysis

The growth phenotype of the *ntrC* mutant was assessed using 96-well microtiter PM3 plates (Biolog, Hayward CA), with each well containing the defined medium with a unique nitrogen (PM3) compound plus an indicator dye for cell respiration. Excluding a carbon-free well (negative control) for each plate, the PM3 assay assessed the ability of a bacterium to utilize 95 nitrogen compounds as the sole nitrogen source (succinate is the carbon source). Experiments were performed following the manufacturer’s instructions. A total of 100 mL of this cell suspension inoculated into the Biolog inoculating fluid was transferred into each well and incubated at 30 °C for 24 h. The colour intensity was measured every 15 min using the OmniLog reader.

### Abiotic stress-resistance assays

Wild-type A1501, *ntrC* mutant A1511 and complemented strain A1512 were grown in LB medium at 30 °C to an OD_600_ of 0.6 and then transferred into fresh LB medium in the presence or absence of 0.5 mM CHP. At the time indicated (oxidative stress, 10 min), 10-fold serial dilutions were performed, and 8 µL of each dilution was spotted onto solid LB plates. These plates were incubated at 30 °C for 24 h before colony growth was observed and enumerated.

## Results

### **Growth analysis of a*P. stutzeri*A1501*ntrC*mutant and a complemented derivative under different nitrogen sources**

The genomic sequence analysis showed that *P. stutzeri* A1501 contains a single copy of a putative *ntrC*-like gene (*PST0349*), which has an open reading frame (ORF) of 1436 bp (Yan et al. 2008). In other bacteria, the *ntrC* gene is located in an operon downstream of the gene coding for its potential sensor kinase NtrB and the gene *glnA*, which encodes a glutamine synthetase (Liu et al. 2017). Similar to other bacteria, the *ntrC*-like gene in *P. stutzeri* A1501 is located downstream of NtrB; however, it is distant from the *glnA* gene. *P. stutzeri* has only one gene that codes for a PII homologue, whereas enterobacteria have two paralogues that code for GlnK and GlnB (van Heeswijk et al. 1996). Further phylogenetic analysis indicated that the product of *ntrC* was highly conserved in Pseudomonas species, and compared with the nitrogen-fixing bacteria, the deduced amino acid sequence of NtrC of A1501 showed the highest similarity (86% identity) to the *A. vinelandii* DJ NtrC protein; however, it only had 45% similarity to the homologous protein of *A. brasilense* (Fig. S1).

**Fig. 1:**
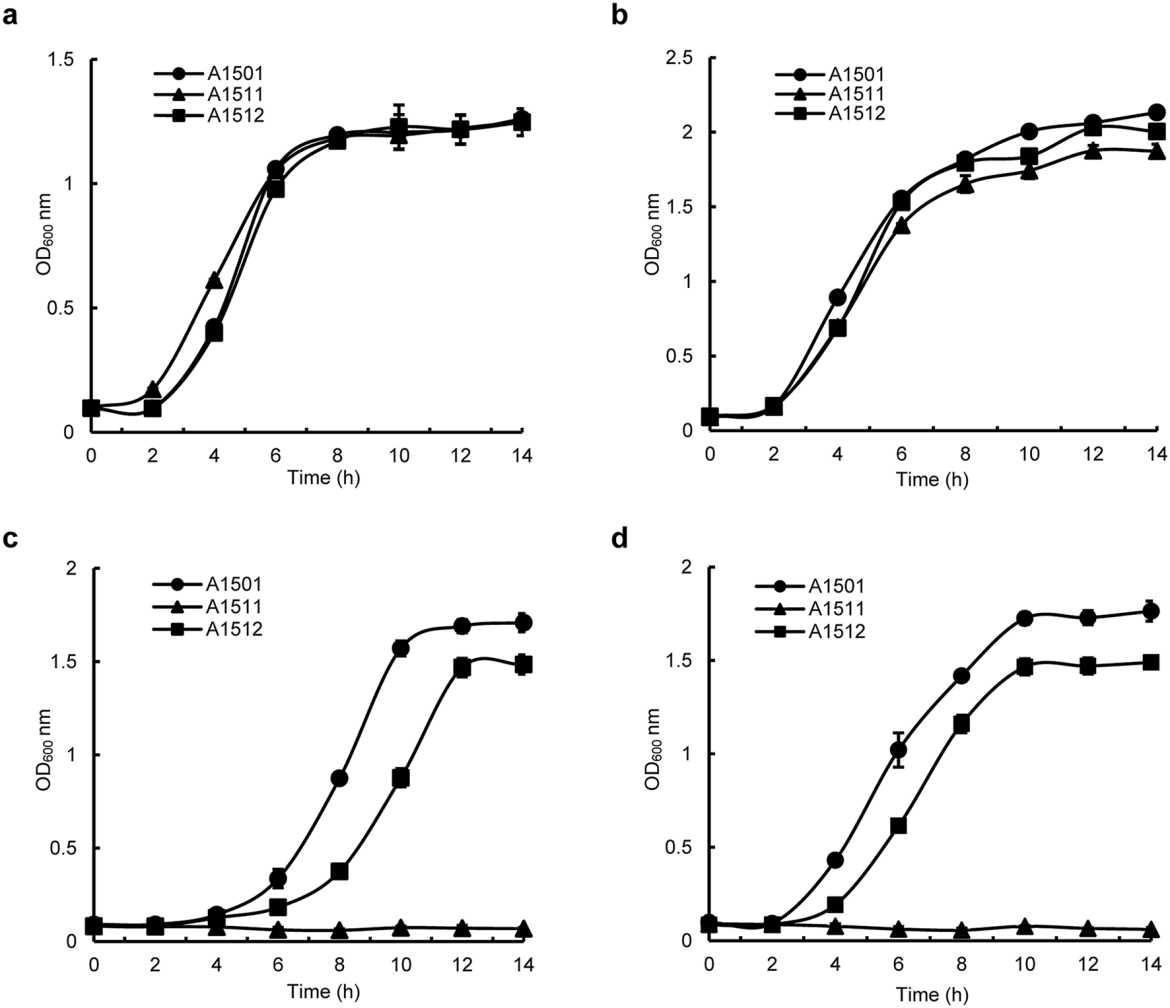
Growth of the wild-type *P. stutzeri* A1501 (●), *ntrC* mutant strain A1511 (▲) and functional complement strain A1512 (■) on ammonium sulfate (**a**), L-serine (**b**), nitrate (**c**) or urea (**d**) as the sole source of nitrogen. Growth was measured in K medium supplemented with ammonium sulfate, L-serine, nitrate or urea (10 mmol·L^−1^) as the sole nitrogen source. The results are the means and standard errors of three independent cultures

**Fig. 2:**
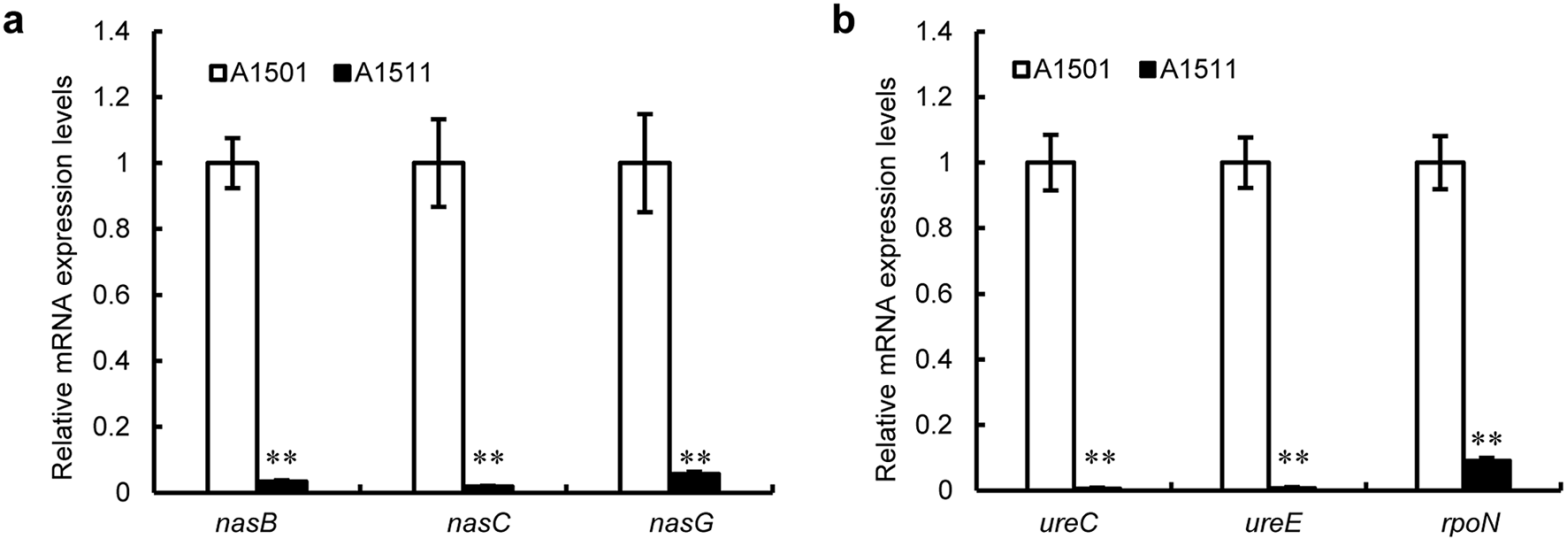
Effect of *ntrC* deletion on the expression of nitrate assimilation-related genes (**a**) and urea catabolism-related genes (**b**). Relative levels of transcripts are presented as the mean values ± standard deviations (SDs) calculated from three sets of independent experiments and normalized to levels in the wild-type strain. The statistical significance of the difference was confirmed by t tests (**P < 0.01)

In bacteria, NtrC was verified as the regulatory player in nitrogen metabolism (Yeom et al. 2010; Kukolj et al. 2020). To study the role of NtrC in A1501, a mutant strain carrying a deletion of the *ntrC* gene, which was designated A1511, and the functional complement strain, which was designated A1512, were constructed (see methods). While the *ntrC* mutant grew at a similar rate and to the same final optical density as the wild type in minimal medium containing ammonium sulfate or serine as the sole nitrogen source and sodium lactate as the carbon source, the mutant lost the utilization capacity for nitrate and urea. The complemented *ntrC* mutant (A1512) was able to reach a final optical density (OD_600_) similar to that of the wild type (Fig. 1). Our qRT–PCR results showed that when mutant A1511 cells were grown in minimal medium containing nitrate or urea as the sole nitrogen source, the transcription of nitrate assimilatory genes (*nasB*, *nasC* and *nasG*) was significantly reduced compared to that of the wild-type strain. The transcriptional levels of the urease accessory protein-encoding gene *ureE* and urease-encoding gene *ureC* were also strongly repressed in the *ntrC* mutant (Fig. 2). Meanwhile, the NtrC-putative binding site was found in the promoter region of *nasB* or *ureE* by bioinformatics analysis (Table S2), suggesting that NtrC might positively regulate nitrate assimilation and urea metabolism of A1501 in a direct manner.

### NtrC affects the metabolic activity of alternative nitrogen sources

**Fig. 3:**
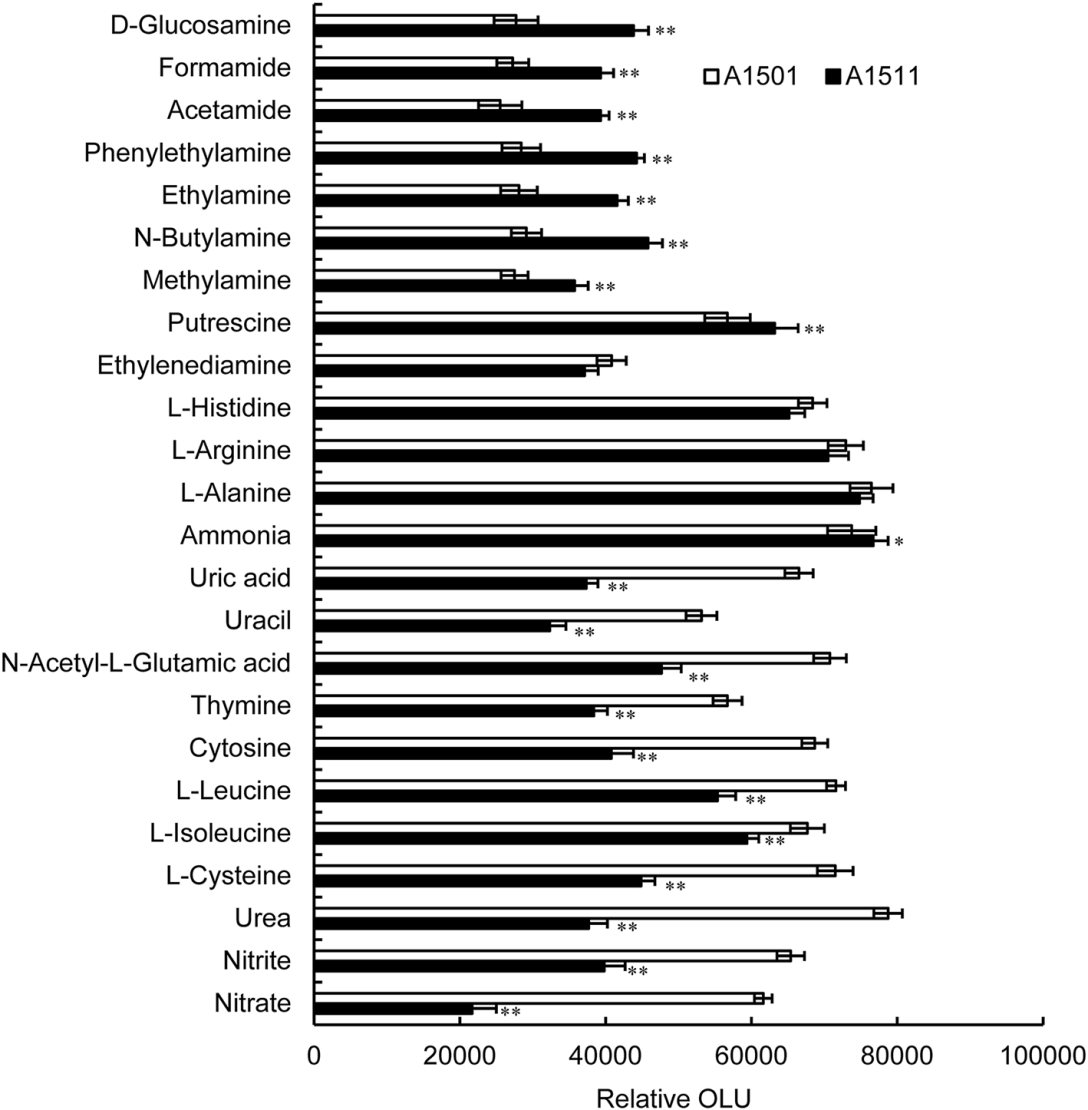
Role of the *ntrC* gene in the utilization of nitrogen substrates as determined by Biolog phenotype microarray (PM3) analysis. Signal intensities were measured using an OmniLog detection system and expressed as relative OmniLog units (OLU). Data are the means ± the standard error from at least three independent cultures, assayed in triplicate. The statistical significance of the difference was confirmed by t tests (**P < 0.01; *P < 0.05)

To further understand the physiological function of NtrC in nitrogen catabolism, the ability of the wild-type strain and the *ntrC* mutant to utilize 95 different nitrogen sources was examined using Biolog Phenotype Microarray (PM) assays. The utilization of 24 N sources was found to be affected by *ntrC* deletion. In 11 cases (nitrate, nitrite, urea, L-cysteine, L-isoleucine, L-leucine, cytosine, thymine, N-acetyl-L-glutamic acid, uracil and uric acid), the *ntrC* mutant was compromised significantly in terms of substrate utilization. In contrast, with 9 various amines (D-glucosamine, formamide, acetamide, phenylethylamine, ethylamine, N-butylamine, methylamine, putrescine and ammonia), the *ntrC* mutant showed enhanced metabolic activity compared to the wild-type (Fig. 3). The inability of mutant A1511 to utilize nitrate as a sole nitrogen source suggests that NtrC controls the expression of genes essential for the assimilation of nitrate. Denitrification is one of the most important processes in the bacterial nitrogen cycle. A1501 can use nitrate as an electron acceptor and shows nitrogen fixation activity under anaerobic conditions (Lin and You 1987). To study the role of NtrC in nitrate respiration, we investigated the denitrification ability of the wild-type A1501 and *ntrC* mutant A1511 under anoxic conditions. The results shown in Fig. S2 confirmed that the ability of mutant A1511 to use nitrate as a terminal electron acceptor was decreased by 70% compared with that of the WT under anoxic conditions. These results suggest that NtrC is essential for nitrogen source utilization under aerobiotic or anoxic conditions.

#### NtrC is required for the positive regulation of nitrogen fixation

**Fig. 4:**
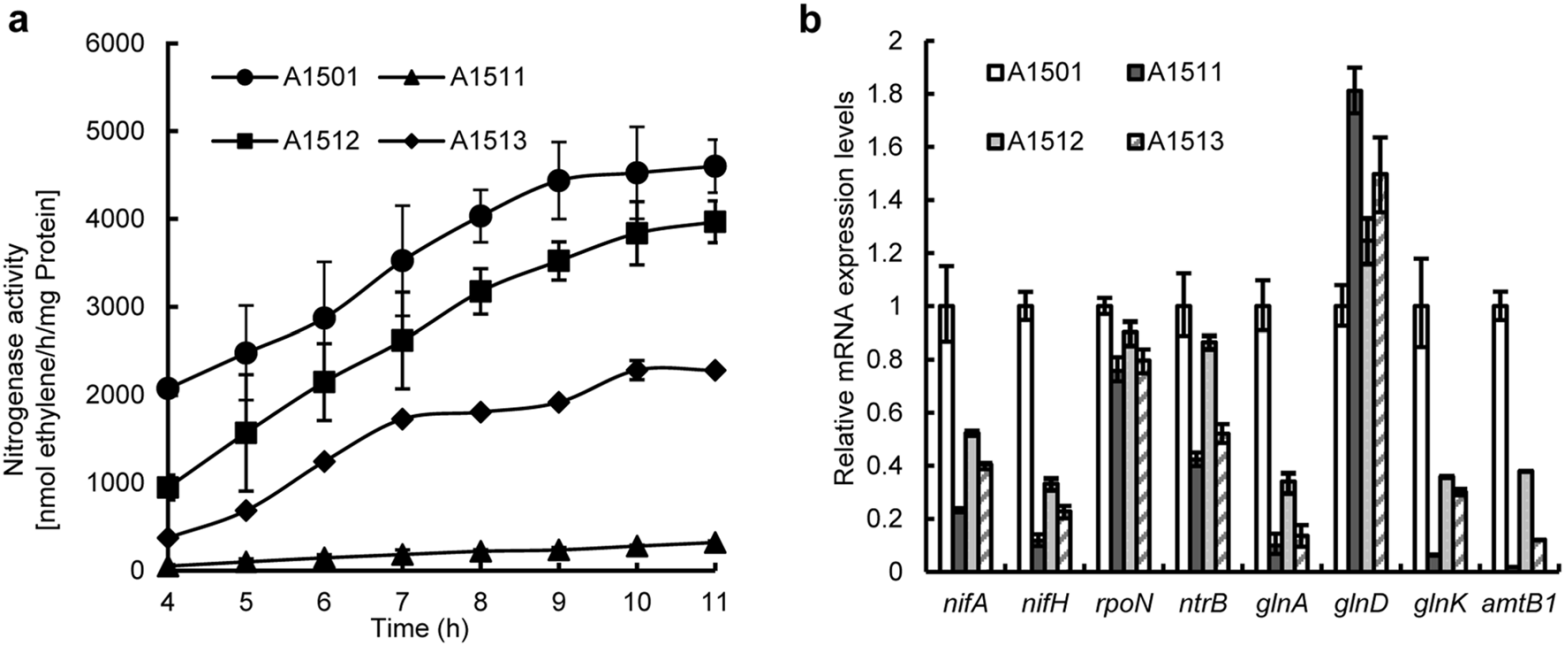
NtrC is required for the positive regulation of nitrogen fixation. **a** Nitrogenase activity in the wild-type A1501 (circles), ∆*ntrC* A1511 (triangles) and the complemented strains A1512 (squares), A1513 (diamond). **b** Effect of *ntrC* deletion on the expression of *nif* genes and their regulators. Relative levels of transcripts are presented as the mean values ± standard deviations (SDs) calculated from three sets of independent experiments and normalized to levels in the wild-type strain

NtrC regulates the function of the nitrogenase-specific regulator NifA in *K. pneumoniae*, although it has no effect on the expression of the nitrogenase complex in *A. brasilense* and *A. vinelandii* (Zhang et al. 1997; Wang et al. 2012). To evaluate the role of NtrC in the nitrogenase system of A1501, nitrogen-fixing activity was detected under nitrogen fixation (nitrogen-free and microaerobic) conditions. *ntrC* deletion resulted in an approximately 90% reduction in nitrogen-fixing activity, and this defect was restored by the introduction of a single copy of *ntrC* (Fig. 4a). The quantitative real-time PCR (qRT–PCR) results showed that the expression levels of the encoding genes for nitrogenase reductase NifH, nitrogenase specific regulator NifA, nitrogen regulatory PII protein GlnK, ammonium transporter AmtB and glutamine synthetase GlnA were decreased to various extents in the *ntrC* mutant compared with the wild type, whereas these inductions were fully or partially restored to wild-type levels by the complementation plasmid with a wild-type *ntrC* gene (Fig. 4b). Furthermore, the conserved putative NtrC-binding site sequence was found in the promoter region of *nifA*, *glnK* or *glnA*, suggesting that their expression might be transcriptionally activated by NtrC and that NtrC positively regulated the nitrogen fixation of *P. stutzeri* A1501 (Table S2).

The two-component systems CbrAB and NtrBC form a network to control the C/N balance in *P. aeruginosa* (Li and Lu 2007). We found that the complementary strain A1513 (mutant A1511 containing pLAcbrB) could recover the inhibited nitrogenase activity caused by *ntrC* deletion (Fig. 4a). The results of quantitative real-time PCR (qRT–PCR) showed that the expression levels of nitrogen fixation-related genes were fully or partially restored to wild-type levels by the complementation plasmid with the *cbrB* gene (Fig. 4b), and this finding strongly indicated that CbrB and NtrC regulate nitrogen fixation in a cooperative manner.

### **Genome-wide analysis of the NtrC regulatory network in*P. stutzeri*A1501 under nitrogen fixation conditions**

To further identify genes that respond to nitrogen fixation conditions in an NtrC-dependent manner, a global transcriptional profiling analysis was conducted with wild-type A1501 and the null-*ntrC* mutant A1511 under nitrogen fixation conditions. Compared to the wild type, the expression levels of a total of 1431 genes exhibited more than a twofold change in the mutant A1511 strain under nitrogen fixation conditions. Among these genes, the transcription of 1253 genes was enhanced, and the expression of 178 genes was repressed in the *ntrC* mutant (DESeq analysis P < 0.05 and fold change > 2.0 or < 0.5). In particular, among these downregulated genes, the 49 kb expression island containing *nif* and other associated genes was markedly downregulated by *ntrC* inactivation, indicating the dominant role of NtrC in the nitrogen fixation regulation of *P. stutzeri* A1501, and these findings are consistent with the phenotypic and expressional analysis described above, thus indicating the reliability of RNA-Seq.

**Fig. 5:**
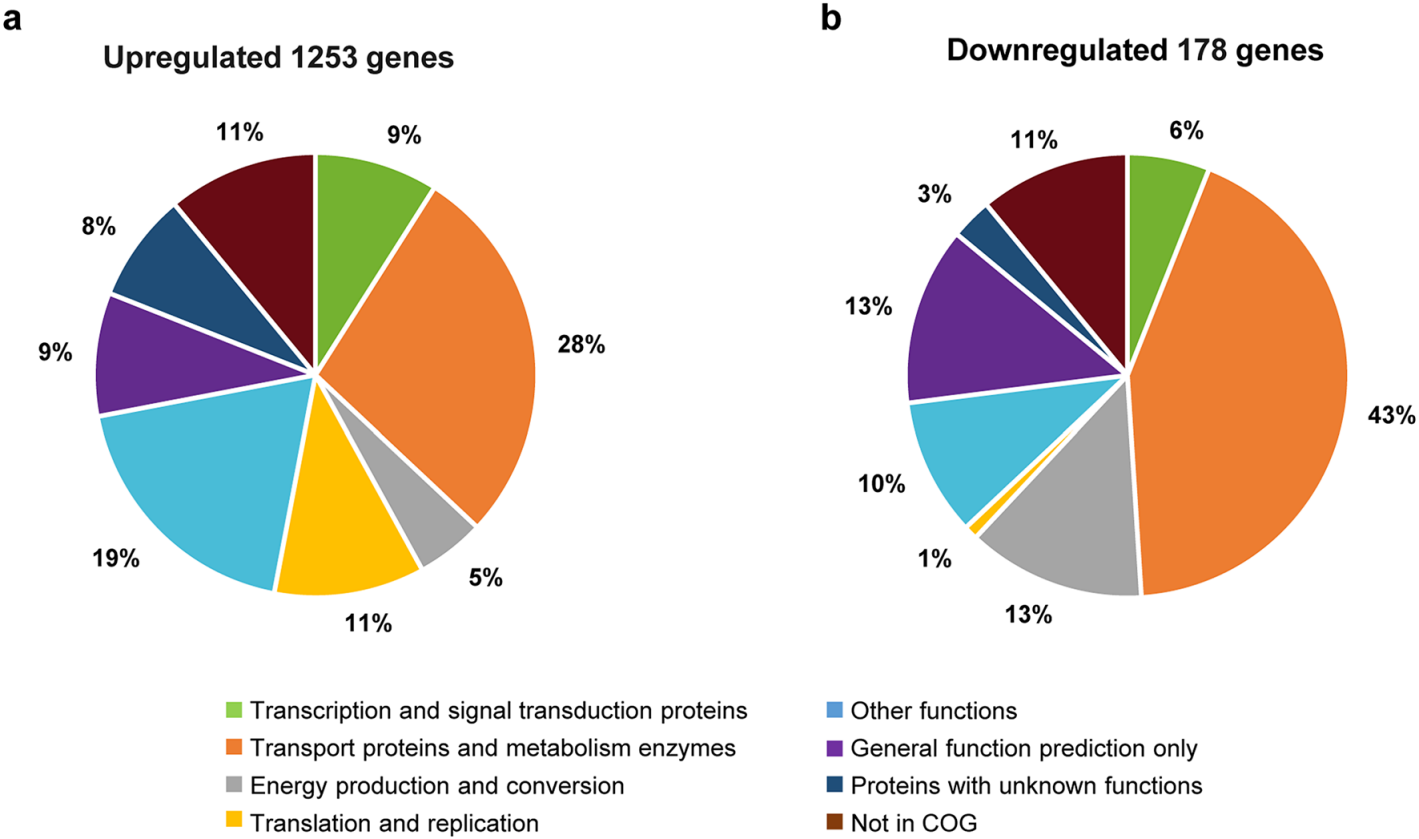
Overview of expression profiling analysis. **a** Functional categories of the core subset of upregulated genes (P < 0.05 and fold change > 2) in the *ntrC* mutant versus wild type under nitrogen fixation conditions. **b** Functional categories of the core subset of downregulated genes (P < 0.05 and fold change < 0.5) in the *ntrC* mutant versus wild type under nitrogen fixation conditions. The percentage of genes in each section is depicted

The *ntrC* mutant resulted in changes in gene expression for several functional categories under nitrogen fixation conditions. These altered genes were further classified according to the COG functional classification system, and the relative occurrence of genes belonging to each category is shown in Fig. 5. Most interestingly, the strong downregulation of genes involved in transport and metabolism enzyme functions (43%) indicated that the deletion of *ntrC* altered the composition of proteins related to the transport and catabolism of nitrogenous compounds. Furthermore, genes related to energy production and conversion (5%) were upregulated, suggesting that the *ntrC* mutant might affect the biosynthetic capabilities of the cell under nitrogen fixation conditions.

Next, to investigate the potential targets of NtrC involved in nitrogen metabolism, the promoter regions of the 1431 changed genes were analysed. The results showed that 147 NtrC-dependent genes exhibited putative NtrC-binding sites, which contain highly conserved GC and GC elements with an 11-nucleotide spacing, by WebLogo analysis (Fig. S3). Among the 756 top-ranking differentially expressed genes (P < 10^−2^, fold change > 2.0 or < 0.5), 141 genes were dramatically repressed in the *ntrC* mutant. The *ntrC* null mutant resulted in genes involved in nitrogen assimilation and nitrogen fixation, such as glutamine synthetase (*glnA*, *PST0353*), PII sensor proteins (*glnK*, *PST0502*) and nitrogen fixation regulatory proteins (*nifA*, *PST1313*; *nifL*, *PST1314*), which showed 0.06-, 0.14-, 0.16- and 0.13-fold reduced transcription, respectively (Table S2). Consistent with the inability of the *ntrC* mutant to grow with urea or nitrate as the sole nitrogen source, the genes required for urea (*ureD-2*, *ureE*, *ureF-2*, *ureG* and *ureA*) and nitrate (*nasS*, *nasT*, *nasA*, *nasF*, *nasD* and *nasB*) transport and utilization displayed strongly downregulated transcription. Additionally, the genes coding for electron transport (*rnfABCDGEH*) and ammonium transporter (*amtB1* and *amtB2*) were significantly repressed in the *ntrC* mutant. Since *amtB1* and *amtB2* are cotranscribed with *glnK*, which has an NtrC-binding site in the promoter region, we inferred that the transcription of *amtB1* and *amtB2* is NtrC-dependent. We also found that the transcription of the genes *ureE* (encoding urease), *nasB* (encoding nitrite reductase), *nasF* (encoding nitrate transporter) and *rnfA* (encoding electron transporter) is NtrC-dependent and has a putative NtrC-binding site in the promoter region, indicating that these genes may be the key genes under NtrC control for nitrate assimilation and urea catabolism. Additionally, the transcription of several genes (*PST2280*, *PST2508* and *PST4035*) involved in chemotaxis was decreased in the *ntrC* mutant; in particular, the putative NtrC-binding site was found in the promoter of *PST2280*, which codes for methyl-accepting chemotaxis receptor proteins (MCPs), and *PST2508*, which codes for methyl-accepting chemotaxis transducers. Chemotaxis is directed motility used by microbes, which sense chemical cues and relocate towards more favourable environments. Since MCPs are the most common receptors in bacteria, we inferred that NtrC might contribute to the interaction of A1501 with plant hosts. Among the 615 top-ranking genes with dramatically increased transcription, the expression levels of several genes involved in the glycolytic pathway were enhanced significantly in the *ntrC* mutant, including *PST0991*, which codes for glucose dehydrogenase; *sucD*, which codes for succinyl-CoA synthetase; *PST3494*, which codes for probable glyceraldehyde-3-phosphate dehydrogenase; *eda-1*, which codes for 4-hydroxy-2-oxoglutarate aldolase; *glk-1* and *glk-2*, which codes for glucokinase; *PST3496*, which codes for 6-phosphogluconolactonase; *PST3497*, which codes for glucose-6-phosphate 1-dehydrogenase; and *PST3500*, which codes for 6-phosphogluconate dehydratase, which showed 3.1-, 2.9-, 6.2-, 9.7-, 3.0-, 5.3-, 4.8-, 6.1- and 11.2-fold increases, respectively (Table S2). Based on these data, we define NtrC as the master nitrogen regulator and infer that it not only activates pathways for nitrogen fixation but also represses carbon catabolism under nitrogen fixation conditions, possibly to prevent excessive carbon and energy flow in the cell.

### **The*ntrC*mutant shows altered oxidative stress response**

The oxygen concentration is one of the main environmental factors affecting biological nitrogen fixation due to the extreme oxygen sensitivity of nitrogenase. To directly test whether the *ntrC* mutant displayed altered resistance to oxidative stress, we compared the growth of wild-type strain A1501, the *ntrC* mutant A1511 and the complementary strain A1512 under oxidative stress conditions by the addition of the oxidizing agent cumene hydroperoxide (CHP). As shown in Fig. S4a, both A1511 and A1512 displayed growth rates similar to that of the wild-type strain in LB medium, indicating that deletion of the *ntrC* gene had no effect on bacterial survival under normal growth conditions. However, we found that the deletion of *ntrC* resulted in significantly increased growth in the presence of 0.5 mM CHP, and the complementary strain recovered the growth capacity to the wild-type level under the same treatment (Fig. S4a). Consistent with the observations mentioned above, oxidative stress-related genes were increased to various extents in the *ntrC* mutant compared with the wild type (Fig. S4b), especially the catalase-encoding gene *katB*, whose expression was increased 11-fold. Bioinformatic analysis revealed one NtrC-binding site in the *katB* promoter region, and we inferred that *katB* is the target gene of NtrC involved in directly regulating optimal oxidative stress resistance.

## Discussion

In this study, we used global gene expression and phenotypic analyses to characterize the role of NtrC in the nitrogen metabolism of *P. stutzeri* A1501 and found that 1431 genes were significantly differentially expressed altered by the *ntrC* mutant. This large number of differentially expressed genes (33.95% of the genome) shows that a major NtrC-dependent transcriptomic response is initiated by *P. stutzeri* A1501 under nitrogen fixation conditions. As expected, genes that are known or predicted to be involved in nitrogen metabolism form the majority of the NtrC regulon. In particular, *nifA*, which codes for the transcriptional activator of all *nif* operons (Chengtao et al. 2004; Demtröder et al. 2019), showed a 0.16-fold decrease, and *glnK*, which codes for a PII family protein (Xu et al. 1998; Blauwkamp and Ninfa 2002), showed a 0.14-fold decrease in *the ntrC* mutant. In *P. stutzeri*, GlnK is required for both NifA synthesis and activity, particularly by preventing the inhibitory effect of NifL on NifA activity (Xie et al. 2006; He et al. 2008). These data were consistent with the observation that inactivation of NtrC affected nitrogenase activity, suggesting a role in positive regulation of *nif* genes. Homologues of the *ntrC* genes have been found in many nitrogen-fixing bacteria, and their roles in nitrogen fixation have been best characterized in *K. pneumoniae*. In this organism, NtrC plays an important role in the transcription of *nifLA* regulatory genes, with NifA activating the transcription of other *nif* operons (Merrick 1983). However, in some diazotrophs, such as *A*. *vinelandii*, *B*. * japonicum*, and *A. brasilense*, NtrC is not necessary for *nif* gene expression. In this study, although NtrC acted as a transcriptional activator of *nifA*, the mutant was Nif^+^, and its nitrogen fixing activity was far lower than that of the wild type. NtrC may positively regulate nitrogen fixation, and the CbrB protein may be able to substitute NtrC to maintain nitrogenase activity.

The largest category of genes in the NtrC regulon is the nitrogen scavenging category, which is logical from an evolutionary perspective because the soil-dwelling *P. stutzeri* A501 encounters various nitrogen sources in the environment and must compete with other soil microbes for nutrients. The genes encoding nitrogen transporters and binding proteins, ammonium transporters, uptake systems for nitrate/nitrite, urea, and amino acids/peptides were all upregulated by NtrC in *P. stutzeri* A1501 under nitrogen fixation conditions. A similar situation is observed for nitrate/nitrite uptake and assimilation because *P. stutzeri* A501 contains two nitrate transporters, binding and response proteins (i.e., PST2003, NasA, NasF, NasE and NasD) and nitrite reductase (i.e., NasB and NasC), which are all upregulated by NtrC in *P. stutzeri* A1501. Assimilatory nitrate reduction to ammonium is a two-step process that includes the reduction of nitrate to nitrite by nitrate reductase followed by the reduction of nitrite to ammonium by nitrite reductase. As confirmed in this study, the assimilatory nitrite reductase NasBC (PST2409 and PST2410) is upregulated by NtrC under nitrogen fixation conditions; however, the nitrate reductase enzyme NasG (PST2411) is not. Therefore, the uptake and assimilation of nitrite, not nitrate, appears to be an important nitrogen stress response in *P. stutzeri* A501. Our study also identified a NtrC-regulated response regulator, NasT (PST2401). In our previous study, *nasT* mutant was unchanged the nitrate uptake capacity of *P. stutzeri* A1501 but could not grow using nitrate as the nitrogen source (unpublished data). However, the precise role of this regulator and nitrate/nitrite respiration in the nitrogen stress response in *P. stutzeri* A1501 requires further investigation.

The phenotype of the *ntrC* mutant indicated that NtrC was not only absolutely required for nitrogen metabolism in *P. stutzeri* but also related to optimal resistance to oxidative stress. The *ntrC* mutant showed significant upregulation of oxidative stress response genes, especially *katB*, which is the most pivotal enzyme for the oxidizing agent CHP (Manso et al. 2020). The bioinformatics analysis revealed one NtrC binding site in the promoter region of *katB*, thus indicating that KatB is a potential target regulated by NtrC. Because oxidative stress is a crucial problem in the survival of nitrogen-fixing bacteria, we inferred that NtrC might regulate oxidative stress resistance via the direct transcriptional activation of *katB*. In this work, NtrC was shown to be involved in regulating the consumption of some nitrogenous compounds. When it was inactivated and subsequently lost function, the ability to utilize some nitrogenous compounds by *P. stutzeri* was impaired. These results further confirmed that the *ntrC* gene was a regulator of the metabolism and assimilation of some nitrogen sources in *P. stutzeri*. However, not all metabolic pathways for nitrogenous compounds were related to NtrC. For example, the *ntrC* mutant could grow well with (NH_4_)_2_SO_4_ and some amino acids as the nitrogen source (Glu and Gln). However, the two amino acids were the key signalling molecules in the nitrogen metabolism pathway and switched to nitrogen assimilation in bacteria. Further studies are required to clarify the NtrC-based mechanisms underlying the response of this bacterium to nitrogen signalling and oxidative stress at the cellular and molecular levels.

Taken together, the results of this study provide a framework for understanding the transcriptional changes of numerous key genes related to various nitrogen metabolism processes controlled by NtrC in *P. stutzeri* A1501 under nitrogen fixation conditions. Chief among these differentially expressed genes are those involved in nitrogen fixation, amino acid catabolism, assimilatory nitrate and ammonium transport. By combining the transcriptome data with bioinformatics analyses, some potential new target genes responsible for electron transport and oxidative stress response regulated by NtrC were discovered, which would help enhance the knowledge of NtrC-based mechanisms underlying both nitrogen metabolism and the environmental adaptation network in *P. stutzeri* A1501.

## Supplementary Information

Below is the link to the electronic supplementary material.

Supplementary material 1 (DOCX 662.6 kb)
