# Supplementary material for: Master regulator NtrC controls the utilization of alternative nitrogen sources in Pseudomonas stutzeri A1501
Source: World J Microbiol Biotechnol. 2021 Sep 15;37(10):177. doi: 10.1007/s11274-021-03144-w (PMC8443478; doi:10.1007/s11274-021-03144-w)
Supplement: Supplementary file 1 — Supplementary material 1 (DOCX 662.6 kb) [file 11274_2021_3144_MOESM1_ESM.docx]

**Supplementary materials:**

**Table S1 Primers used in this study**

| **Primer^a^** | **Sequence (5’-3**’**)^b^** | **Amplicon size (bp)** | **Purpose** |
| --- | --- | --- | --- |
| upF | TTGGATCCAGCGTGGCATATTCGTG | 772 | A1511 construct |
| upR | GTCTCCATGCGAGCTCGAATTCCTGACGCGCAAGATCAAG |  |  |
| downF | GAAATTCGTCGAAGCTCTATGTCGAAGCTCTGCGTG | 806 |  |
| downR | TTTAAGCTTCCAAGCAGGAAACCACCAAG |  |  |
| CmF | CTTGATCTTGCGCGTCAGGAATTCGAGCTCGCATGGAGAC | 882 |  |
| CmR | CACGCAGAGCTTCGACATAGAGCTTCGACGAATTTC |  |  |
| testF | CGGTATTGGGCGGTATTTCC | 1794 | Validation of A1511 by PCR |
| testR | ACACGCTCTTCTACCCGAT |  |  |
| ntrCF | TTTAAGCTTTGTCGCAGGTCGGAT | 1569 | pLAntrC construct |
| ntrCR | TTGGATCCAGAACATCATCAGTCAG |  |  |
| cbrBF | CGGGATCCTGGAGGACGAAGGCAG | 1716 | pLAcbrB construct |
| cbrBR | ACAAGCTTTTACCCGGCGGCGCTA |  |  |
| RTnifHF | GAGATGATGGCGATGTATGC | 113 | qRT-PCR |
| RTnifHR | GGTCGGTGTTGCGGCTGTTG |  |  |
| RTnifAF | CGCGAAGACCTCTACTACCG | 139 |  |
| RTnifAR | CAGCTTGAGTTTGCGACCCT |  |  |
| RTrpoNF | CGAGTGCCGACAATACCTT | 103 |  |
| RTrpoNR | GGTGGCGACCTTCATCAGT |  |  |
| RTglnAF | CGGAGCCGGAGTTCTTCATC | 110 |  |
| RTglnAR | GACGTCCTGGTCGGTCATCC |  |  |
| RTglnKF | AGTCACTGCCATCATCAAGCC | 183 |  |
| RTglnKR | GCCACGTCGATCTTCACCTTT |  |  |
| RTglnDF | ATCAACGCCACCAACCC | 129 |  |
| RTglnDR | GCGAATCTGCTCCTCACG |  |  |
| RTnasDF | TCGGTGACTGGCTGATGG | 110 |  |
| RTnasDR | CTCGGCTGTCTGCTTTGG |  |  |
| RTnasEF | ATATCTGCGCACTGGATGAG | 165 |  |
| RTnasER | GCTTGCCGTAGACGATCC |  |  |
| RTnasBF | CTATCCGCTGACGCTCAAC | 176 |  |
| RTnasBR | GCCGACTCTTCATCTTCACC |  |  |
| RTureCF | CCTGTTACGGCGGTTCG | 195 |  |
| RTureCR | CCTGATAGTTCTGCGGGTC |  |  |
| RTureEF | CTGCCCAGTCAACTACCACC | 175 |  |
| RTureER | CCCTTCGGGATACAGATGCT |  |  |
| RTkatEF | GCTGGACCCGACCAAAAT | 250 |  |
| RTkatER | CGGACGGTTGATCGGAAT |  |  |
| RTkatAF | ATGGACCAATCTGAAGAGCC | 125 |  |
| RTkatAR | CGTGCATGAACCGATAACC |  |  |
| RTkatGF | TTCCGCAACTACTACCACGAG | 211 |  |
| RTkatGR | TGTCCAGCAGGTTGACGAAG |  |  |
| RTkatBF | CTTCTTGCTGAACGAGCGATAC | 288 |  |
| RTkatBR | TTCTCCTACGCCGATACCCA |  |  |
| RTsodCF | CACGGCTTTCATATCCACG | 227 |  |
| RTsodCR | ATGTCACCCAGGCTCTTCA |  |  |
| RTsodBF | AAGGAAGAGTTCACCAAGACCG | 239 |  |
| RTsodBR | ACGAAGTCCCAGTTCACCAG |  |  |
| RTaphF-1F | ACCGGAAGTCATTGAGCAGA | 223 |  |
| RTaphF-2R | CTCGCCGTTGAGGTAGATGC |  |  |
| RTaphF-2F | CAGGACAGCGAGAAGTAGGTT | 295 |  |
| RTaphF-2R | GCGAGAAGTCCCAGGAAATG |  |  |
| RTaphCF | GTCTTCTCGCCCTCTTTCCA | 109 |  |
| RTaphCR | AGATCGCTCGTGACGTGTCC |  |  |
| RTgstAF | GGAACTGGAGCTGCTGTACG | 136 |  |
| RTgstAR | CTCTGCCAGGGTCAGGTCAC |  |  |

^a^F, forward; R, reverse; ^b^Restriction sites are underlined.

**Table S2** **Description of some NtrC-regulated genes with statistically significant differential expression, comparing the expression profile of the *ntrC* mutant with the profile of the wild type under nitrogen fixation conditions (DE-Seq analysis, p-value < 10^−2^, Fold Change > 2.0 or <0.5)**

| **Class** | **Locus ID^a^** | **Gene name** | **Description** | **Fold change^b^** |
| --- | --- | --- | --- | --- |
| **Downregulated genes**  **Nitrogen fixation** |  |  |  |  |
|  | PST0350 | *ntrB* | nitrogen regulation protein | 0.38 |
|  | PST0353 | *glnA* | glutamine synthetase | 0.06 |
|  | PST0502 | *glnK* | nitrogen regulatory protein P-II | 0.14 |
|  | PST1302 | *PST1302* | glutaredoxin-related protein | 0.12 |
|  | PST1303 | *PST1303* | thiosulfate sulfurtransferase | 0.07 |
|  | PST1304 | *nifQ* | nitrogen fixation protein | 0.10 |
|  | PST1305 | *PST1305* | arsenate reductase related protein | 0.08 |
|  | PST1305a | *fdxN* | ferredoxin-like protein | 0.10 |
|  | PST1306 | *nifB* | FeMo cofactor biosynthesis protein | 0.07 |
|  | PST1313 | *nifA* | nitrogen fixation positive regulatory protein | 0.16 |
|  | PST1314 | *nifL* | nitrogen fixation negative regulatory protein | 0.13 |
|  | PST1315 | *rnfA* | electron transport complex, A subunit | 0.09 |
|  | PST1316 | *rnfB* | electron transport complex, B subunit | 0.10 |
|  | PST1317 | *rnfC* | electron transport complex, C subunit | 0.08 |
|  | PST1318 | *rnfD* | electron transport complex, D subunit | 0.11 |
|  | PST1319 | *rnfG* | electron transport complex, G subunit | 0.12 |
|  | PST1320 | *rnfE* | electron transport complex, E subunit | 0.14 |
|  | PST1321 | *rnfH* | electron transport complex, H subunit | 0.15 |
|  | PST1322 | *nifY2* | dinitrogenase iron-molybdenum cofactor biosynthesis | 0.09 |
|  | PST1323 | *PST1323* | nitrogen fixation-related protein | 0.11 |
|  | PST1324 | *PST1324* | conserved hypothetical protein | 0.10 |
|  | PST1325 | *PST1325* | conserved hypothetical protein | 0.09 |
|  | PST1327 | *nifD* | MoFe protein, alpha subunit | 0.05 |
|  | PST1328 | *nifK* | MoFe protein, beta subunit | 0.05 |
|  | PST1329 | *nifT* | nitrogen fixation protein | 0.06 |
|  | PST1330 | *nifY* | nitrogenase iron-molybdenum cofactor biosynthesis | 0.13 |
|  | PST1331 | *PST1331* | conserved hypothetical protein | 0.08 |
|  | PST1332 | *PST1332* | leucine-rich repeat domain protein | 0.09 |
|  | PST1333 | *nifE* | nitrogenase iron-molybdenum cofactor biosynthesis protein | 0.13 |
|  | PST1334 | *nifN* | nitrogenase iron-molybdenum cofactor biosynthesis protein | 0.12 |
|  | PST1335 | *nifX* | nitrogenase iron-molybdenum cofactor biosynthesis protein | 0.14 |
|  | PST1336 | *PST1336* | protein of unknown function DUF269 | 0.12 |
|  | PST1337 | *PST1337* | protein of unknown function DUF683 | 0.18 |
|  | PST1338 | *PST1338* | ferredoxin, 4Fe-4S | 0.12 |
|  | PST1339 | *PST1339* | ferredoxin, 2Fe-2S | 0.11 |
|  | PST1340 | *PST1340* | conserved hypothetical protein | 0.17 |
|  | PST1341 | *PST1341* | conserved hypothetical protein | 0.15 |
|  | PST1342 | *PST1342* | conserved hypothetical protein | 0.23 |
|  | PST1343 | *PST1343* | conserved hypothetical protein | 0.15 |
|  | PST1344 | *PST1344* | conserved hypothetical protein | 0.13 |
|  | PST1345 | *modC* | molybdenum transport protein | 0.38 |
|  | PST1347 | *modA* | molybdenum ABC transporter | 0.18 |
|  | PST1348 | *PST1348* | putative molybdenum-binding protein | 0.22 |
|  | PST1349 | *hesB* | Fe-S cluster assembly protein | 0.11 |
|  | PST1350 | *nifU* | Fe-S cluster assembly protein | 0.08 |
|  | PST1351 | *nifS* | nitrogenase metalloclusters biosynthesis protein | 0.08 |
|  | PST1352 | *nifV* | homocitrate synthase | 0.11 |
|  | PST1353 | *cysE* | serine acetyltransferase | 0.15 |
|  | PST1354 | *PST1354* | conserved hypothetical protein | 0.16 |
|  | PST1355 | *nifW* | nitrogenase stabilizing/protective protein | 0.09 |
|  | PST1356 | *nifZ* | Fe-S cofactor synthesis protein | 0.14 |
|  | PST1357 | *nifM* | putative a peptidyl-prolyl cis/trans isomerase | 0.15 |
|  | PST1358 | *PST1358* | ATP-dependent Clp protease | 0.14 |
|  | PST1359 | *nifF* | flavodoxin required for electron transfer to the Fe protein | 0.13 |
| **Nitrate assimilation/dissimilation** | PST1955 | *PST1955* | nitrite reductase [NAD(P)H] large subunit | 0.02 |
|  | PST2400 | *nasS* | nitrate-binding protein NasS | 0.20 |
|  | PST2401 | *nasT* | response regulator NasT | 0.22 |
|  | PST2406 | *nasA* | nitrate transporter | 0.04 |
|  | PST2409 | *nasB* | assimilatory nitrite reductase large subunit | 0.03 |
|  | PST4092 | *nasF* | NrtA-type periplasmic nitrate transport binding protein | 0.03 |
|  | PST4094 | *nasD* | nitrate ABC transporter | 0.21 |
|  | PST0503 | *amtB1* | ammonium transporter | 0.06 |
|  | PST0504 | *amtB2* | ammonium transporter | 0.13 |
| **Amino acid / nucleotide metabolism and transport** | PST1993 | *PST1993* | glutamate--ammonia ligase | 0.06 |
|  | PST1994 | *PST1994* | proable homoserine O-acetyltransferase | 0.41 |
|  | PST1995 | *PST1995* | taurine ABC transporter | 0.36 |
|  | PST2066 | *PST2066* | phosphoserine aminotransferase | 0.33 |
|  | PST2197 | *PST2197* | probable acyl-CoA dehydrogenase | 0.21 |
|  | PST2243 | *PST2243* | phosphotransferase enzyme family protein | 0.42 |
|  | PST2244 | *PST2244* | probable short-chain dehydrogenase | 0.38 |
|  | PST2594 | *PST2594* | probable oxidoreductase | 0.31 |
|  | PST2897 | *PST2897* | probable oxidoreductase | 0.12 |
|  | PST3080 | *PST3080* | oxidoreductase, 2OG-Fe (II) oxygenase family | 0.17 |
|  | PST3569 | *codB* | cytosine transporter | 0.29 |
|  | PST3570 | *codA* | cytosine deaminase | 0.42 |
|  | PST3584 | *PST3584* | oxidoreductase, 2OG-Fe (II) oxygenase family | 0.12 |
|  | PST3720 | *PST3720* | branched-chain amino acid ABC transporter, periplasmic amino acid-binding protein, putative | 0.06 |
|  | PST3723 | *PST3723* | branched-chain amino acid ABC transporter | 0.30 |
|  | PST3726 | *ureD-2* | urease accessory protein | 0.07 |
|  | PST3727 | *ureA* | urease, gamma subunit | 0.05 |
|  | PST3736 | *ureE* | urease accessory protein | 0.05 |
|  | PST3737 | *ureF-2* | urease accessory protein | 0.03 |
|  | PST3738 | *ureG* | urease accessory protein | 0.33 |
|  | PST3878 | *PST3878* | probable acyl-CoA dehydrogenase | 0.36 |
| **Carbohydrate transport and mechanism** | PST0432 | *glcE* | glycolate oxidase | 0.35 |
|  | PST2265 | *PST2265* | quinoprotein alcohol dehydrogenase | 0.12 |
|  | PST2270 | *PST2270* | quinoprotein alcohol dehydrogenase | 0.07 |
| **Lipid transport and mechanism** | PST_0685 | *phaB* | 3-oxoacyl-(acyl-carrier-protein) reductase | 0.33 |
|  | PST_1300 | *PST_1300* | alpha-ribazole-5'-phosphate phosphatase | 0.37 |
|  | PST_1301 | *cobS* | cobalamin (5'-phosphate) synthase | 0.33 |
| **Transport** | PST2269 | *PST2269* | periplasmic binding protein, putative | 0.10 |
|  | PST2284 | *PST2284* | ABC efflux transporter | 0.17 |
|  | PST2862 | *PST2862* | nucleoside-binding outer membrane protein | 0.24 |
|  | PST2907 | *PST2907* | ABC transporter | 0.10 |
|  | PST2933 | *PST2933* | ABC transporter substrate-binding protein | 0.24 |
|  | PST3505 | *PST3505* | iron permease | 0.25 |
|  | PST1992 | *PST1992* | ABC transporter | 0.20 |
|  | PST2003 | *PST2003* | ABC-type nitrate/sulfonate/bicarbonate transport systems | 0.11 |
|  | PST2010 | *PST2010* | ABC transporter | 0.15 |
|  | PST2900 | *PST2900* | probable ABC transporter | 0.25 |
| **Defense mechanism** | PST0646 | *PST0646* | type I restriction-modification system, S subunit | 0.00 |
|  | PST0647 | *PST0647* | type I restriction-modification system, M subunit | 0.04 |
|  | PST2272 | *PST2272* | pentapeptide repeat family protein | 0.09 |
| **Cell wall/membrane envelope biogenesis** | PST2283 | *PST2283* | outer membrane protein, putative | 0.16 |
|  | PST3079 | *PST3079* | membrane protein, bmp family | 0.13 |
|  | PST2068 | *PST2068* | coenzyme PQQ synthesis protein E | 0.14 |
|  | PST2067 | *PST2067* | predicted membrane protein | 0.16 |
| **Transcription and signal transduction** | PST1804 | *PST1804* | sensory box/GGDEF family protein | 0.21 |
|  | PST1991 | *ttgR* | transcriptional regulator | 0.28 |
|  | PST1981 | *PST1981* | probable two-component sensor | 0.05 |
|  | PST2277 | *PST2277* | DNA-binding response regulator, LuxR family | 0.27 |
|  | PST2278 | *PST2278* | sensor histidine kinase | 0.37 |
|  | PST2048 | *PST2048* | universal stress protein family | 0.34 |
| **Cell motility** | PST2280 | *PST2280* | methyl-accepting chemotaxis receptor/sensory transducer | 0.07 |
|  | PST2508 | *PST2508* | methyl-accepting chemotaxis transducer | 0.10 |
|  | PST4035 | *PST4035* | methyl-accepting chemotaxis protein | 0.09 |
| **Others** | PST0103 | *PST0103* | predicted alpha/beta hydrolase | 0.30 |
|  | PST0297 | *PST0297* | conserved hypothetical protein | 0.30 |
|  | PST1715 | *PST1715* | TldD/PmbA family protein | 0.13 |
|  | PST1980 | *PST1980* | conserved hypothetical protein | 0.11 |
|  | PST1998 | *PST1998* | chain F, crystal structure of creatinine amidohydrolase | 0.15 |
|  | PST2002 | *PST2002* | conserved hypothetical protein | 0.10 |
|  | PST2069 | *PST2069* | pyrroloquinoline quinone biosynthesis protein D | 0.11 |
|  | PST2071 | *PST2071* | probable aldehyde dehydrogenase | 0.10 |
|  | PST2267 | *PST2267* | hydrolase, putative | 0.42 |
|  | PST2268 | *PST2268* | conserved hypothetical protein | 0.04 |
|  | PST2271 | *exaB* | cytochrome c550 | 0.17 |
|  | PST2279 | *PST2279* | uncharacterized copper-binding protein | 0.29 |
|  | PST2281 | *PST2281* | conserved hypothetical protein | 0.16 |
|  | PST2282 | *PST2282* | conserved hypothetical protein | 0.28 |
|  | PST2402 | *PST2402* | conserved hypothetical protein | 0.15 |
|  | PST2405 | *PST2405* | conserved hypothetical protein | 0.00 |
|  | PST2898 | *PST2898* | putative alkyl salicylate esterase | 0.29 |
|  | PST2899 | *PST2899* | hypothetical protein | 0.08 |
|  | PST3129 | *PST3129* | conserved hypothetical protein | 0.18 |
|  | PST3566 | *cynS* | cyanate lyase | 0.20 |
|  | PST3597 | *PST3597* | bacterial luciferase family protein | 0.06 |
|  | PST3598 | *PST3598* | isochorismatase family protein | 0.08 |
|  | PST3790 | *PST3790* | MaoC domain protein | 0.28 |
|  | PST4076 | *PST4076* | probable oxidoreductase | 0.32 |
| **Upregulated genes** |  |  |  |  |
| **Glycolysis** | PST0991 | *PST0991* | glucose dehydrogenase | 3.13 |
|  | PST1879 | *sucD* | succinyl-CoA synthetase alpha chain | 2.85 |
|  | PST2443 | *glk-1* | glucokinase | 3.00 |
|  | PST3494 | *PST3494* | probable glyceraldehyde-3-phosphate dehydrogenase | 6.21 |
|  | PST3495 | *eda-1* | 4-hydroxy-2-oxoglutarate aldolase | 9.70 |
|  | PST3496 | *PST3496* | 6-phosphogluconolactonase | 4.83 |
|  | PST3497 | *PST3497* | glucose-6-phosphate 1-dehydrogenase | 6.16 |
|  | PST3499 | *glk-2* | glucokinase | 5.15 |
|  | PST3500 | *PST3500* | 6-phosphogluconate dehydratase | 11.16 |

^a^ An underlined gene identifier (ID) indicates that its promoter region contains the putative NtrC-binding site; ^b^ Fold change (FC) of transcription, comparing *ntrC* mutant A1511 with wild type A1501 grown under nitrogen fixation conditions.

**
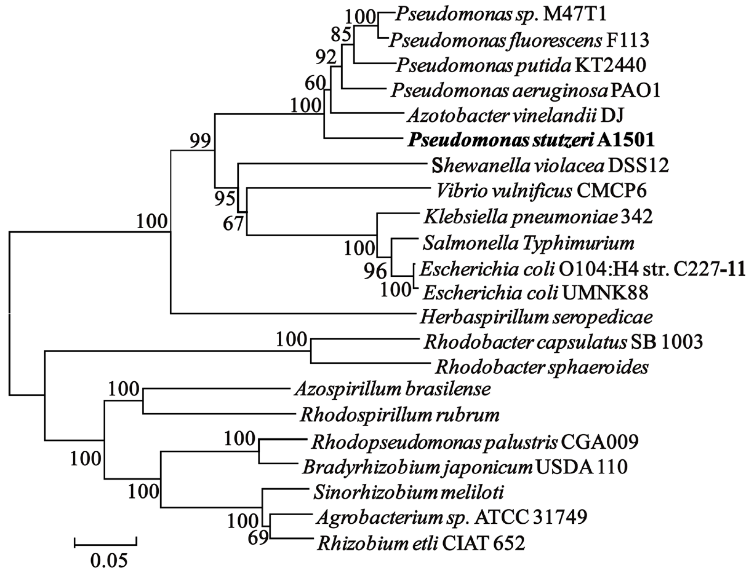
**

**Fig. S1** Unrooted neighbour-joining phylogenetic tree of the A1501 NtrC and related proteins from other bacteria. The percentage of trees from 1000 bootstrap resamples supporting the topology is indicated when above 50. The NtrC protein of *P. stutzeri* A1501 is indicated with black bold font.


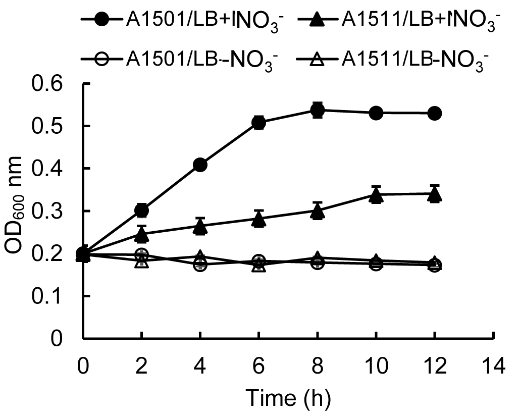


**Fig. S2** Growth of the WT A1501 (circles) and ∆*ntrC* A1511 (triangles) cells under anoxic conditions without nitrate (white symbols) and with nitrate (black symbols). The results are the means and standard errors of three independent cultures.

**
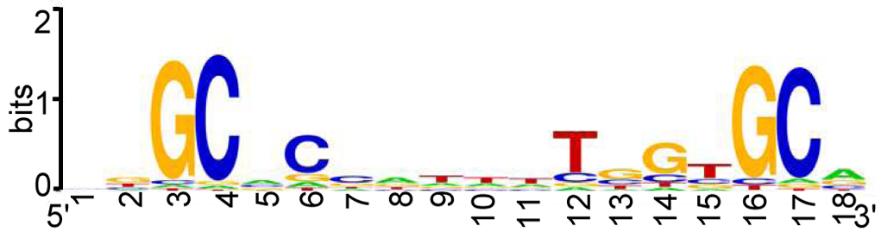
**

**Fig. S3** WebLogo analysis showed that the NtrC putative binding sites in the promoter regions of the NtrC-dependent genes were relatively conserved in *P. stutzeri* A1501.


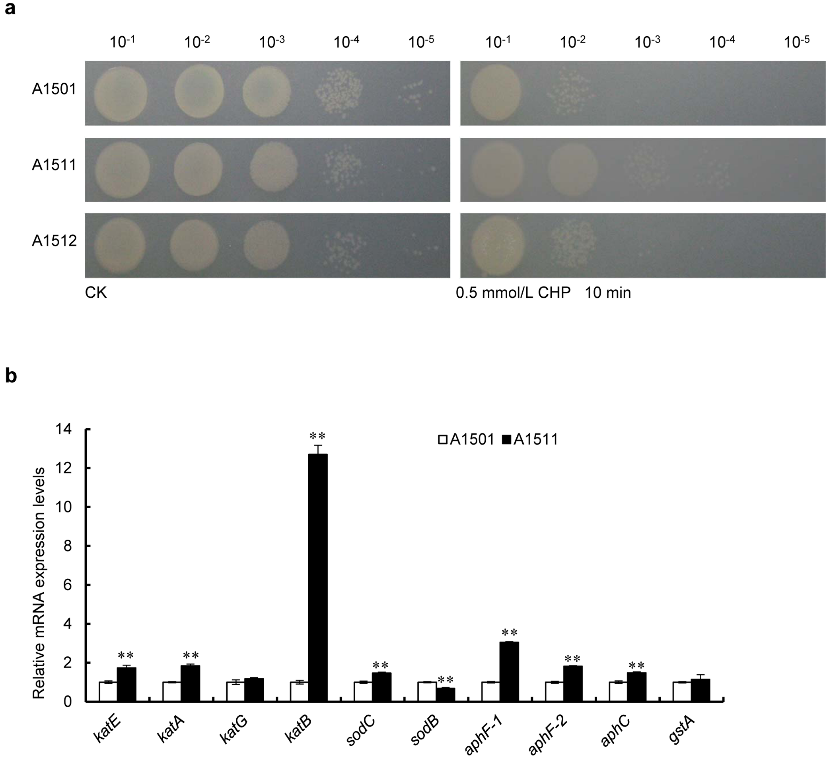


**Fig. S4** Effect of *ntrC* deletion on the response to oxidative stress in *P. stutzeri* A15101. (a) Survival phenotype plate assay with oxidative stress. Serial 10-fold dilutions of OD-standardized cultures were spotted on LB plates after exposure to 0.5 mM CHP. A1501, wild type; A1511, Δ*ntrC* mutant; A1512, complemented strain; CK, untreated culture control. (b) Effect of the *ntrC* mutation on the expression of oxidative stress-related genes. Relative levels of transcripts are presented as the mean values ± standard deviations (SDs) calculated from three sets of independent experiments and normalized to levels in the wild-type strain. The statistical significance of the difference was confirmed by t tests (**, P<0.01).
